# Multi-Target Modulation of Metabolic and Steroidogenic Pathways by *Cinnamomum burmannii* and *Myristica fragrans* in Polycystic Ovary Syndrome: An Integrative Transcriptomics, Metabolomic, Pharmacoinformatics and Experimental Validation

**DOI:** 10.3390/nu18081305

**Published:** 2026-04-21

**Authors:** Taruna Ikrar, Salmon Charles Siahaan, Hendy Hendarto, Arifa Mustika, Eighty Mardiyan Kurniawati, Wiskara Jatipradresthya, Edwin Hadinata, Nurpudji Astuti Taslim, Dante Saksono Harbuwono, Raymond Rubianto Tjandrawinata, Fahrul Nurkolis

**Affiliations:** 1 The Indonesian Food and Drug Authority (BPOM), Jakarta 10560, Indonesia; 2 Universiti Teknologi Malaysia (UTM) Kuala Lumpur, Jalan Sultan Yahya Petra, Kuala Lumpur 54100, Wilayah Persekutuan Kuala Lumpur, Malaysia; 3 School of Medicine, Faculty of Medicine, Universitas Ciputra, Surabaya 60219, Indonesia; 4 Department of Anatomy, Histology, and Pharmacology, Faculty of Medicine, Universitas Airlangga, Surabaya 60131, Indonesia; 5 Department of Obstetrics and Gynecology, Faculty of Medicine, Universitas Airlangga, Surabaya 60131, Indonesia; 6 Center for Health Policy and Management, Faculty of Medicine, Public Health and Nursing, Universitas Gadjah Mada, Yogyakarta 55284, Indonesia; 7 Division of Clinical Nutrition, Department of Nutrition, Faculty of Medicine, Hasanuddin University, Makassar 90245, Indonesia; 8 Division of Endocrinology, Metabolism, and Diabetes, Department of Internal Medicine, Faculty of Medicine, Universitas Indonesia, Dr. Cipto Mangunkusumo National Referral Hospital, Jakarta 10430, Indonesia; 9 Center for Pharmaceutical and Nutraceutical Research and Policy, School of Bioscience, Technology, and Innovation, Atma Jaya Catholic University of Indonesia, Jakarta 12930, Indonesia; 10 Institute for Research and Community Service, State Islamic University of Sunan Kalijaga (UIN Sunan Kalijaga), Yogyakarta 55281, Indonesia; 11 Faculty of Medicine, Universitas Airlangga, Surabaya 60131, Indonesia; 12 Medical Research Center of Indonesia, Surabaya 60281, Indonesia

**Keywords:** polycystic ovary syndrome, cinnamon, nutmeg, transcriptomics, network pharmacology, molecular docking, steroidogenesis, insulin signaling, multi-target therapy, functional validation

## Abstract

**Background:** Polycystic ovary syndrome (PCOS) is a complex endocrine–metabolic disorder characterized by interconnected dysregulation of steroidogenesis and insulin signaling. Multi-target therapeutic strategies are increasingly needed to address its heterogeneous pathophysiology. **Methods:** An integrative approach combining transcriptomic analysis of GSE137684, including stratification of normoandrogenic and hyperandrogenic PCOS subtypes to capture androgen-related heterogeneity, network pharmacology, molecular docking, and in vitro validation was employed. Principal component analysis (PCA), differential expression analysis, and enrichment analyses were used to identify candidate genes and pathways. Molecular docking evaluated interactions between phytochemicals from *Cinnamomum burmannii* and *Myristica fragrans* and key PCOS targets. Functional validation was performed in insulin-resistant 3T3-L1 adipocytes and DHEA-induced KGN cells, assessing cell viability, lipid accumulation, glucose uptake, gene expression, and hormone levels. **Results:** PCA revealed partial separation between PCOS and the control samples, with PC1 and PC2 explaining 44.8% and 12.5% of variance, respectively. No genes remained significant after multiple testing correction; however, nominally significant candidates (*p* < 0.01) highlighted pathways related to steroidogenesis and metabolic regulation. Network analysis identified key hub genes including CYP17A1, CYP19A1, AKT1, ESR1, and MAPK1. Molecular docking demonstrated strong binding affinities, with top compounds showing binding energies up to −11.4 kcal/mol (CYP17A1) and −10.9 kcal/mol (AKT1). In vitro, cell viability remained above 80% across all tested concentrations, indicating low cytotoxicity. Treatment significantly reduced lipid accumulation and enhanced glucose uptake in insulin-resistant 3T3-L1 cells (*p* < 0.05). Additionally, expression of AKT1 and MAPK1 was significantly restored (*p* < 0.05). In KGN cells, testosterone levels were significantly decreased while the estradiol levels increased (*p* < 0.05), accompanied by the downregulation of CYP17A1 and upregulation of CYP19A1 (*p* < 0.05). The combination treatment exhibited more consistent effects across metabolic and hormonal endpoints. **Conclusions:** *Cinnamomum burmannii* and *Myristica fragrans* exert multi-target effects on metabolic and steroidogenic pathways relevant to PCOS. This integrative study demonstrates that transcriptomics-guided network pharmacology combined with experimental validation can identify synergistic phytotherapeutic strategies for complex endocrine disorders.

## 1. Introduction

Polycystic ovary syndrome (PCOS) is among the most common endocrine conditions affecting women of reproductive age and is characterized by substantial clinical and molecular heterogeneity [1]. Contemporary diagnostic frameworks recognize PCOS as a spectrum disorder, with varying constellations of hyperandrogenism, ovulatory dysfunction, and polycystic ovarian morphology, often accompanied by cardiometabolic comorbidities [2]. This heterogeneity is not merely descriptive: it reflects a multi-layered pathophysiology where reproductive and metabolic disturbances reinforce one another over time. In particular, insulin resistance and compensatory hyperinsulinemia can amplify androgen excess, while androgen excess can worsen adipose and skeletal muscle insulin sensitivity, creating a self-perpetuating cycle that contributes to persistent symptoms and long-term risk [3].

At the mechanistic level, dysregulated steroidogenesis is central to many PCOS phenotypes and is closely coupled to intracellular signaling pathways that govern cellular metabolism, proliferation, and hormone responsiveness. Key steroidogenic enzymes, including CYP17A1 (critical for androgen biosynthesis) and CYP19A1/aromatase (responsible for conversion of androgens to estrogens), influence the balance between testosterone and estradiol and thereby shape ovarian microenvironment, follicular development, and granulosa cell function [4]. In parallel, signal transduction nodes such as the PI3K–AKT and ERK/MAPK pathways integrate metabolic cues with ovarian responses, while estrogen receptor signaling (notably ESR1) modulates transcriptional programs relevant to folliculogenesis and endocrine homeostasis [5]. In addition to metabolic and hormonal dysregulation, accumulating evidence indicates that oxidative stress and chronic low-grade inflammation play critical roles in the pathophysiology of PCOS [6,7]. Increased reactive oxygen species production, impaired antioxidant defenses, and elevated inflammatory mediators have been linked to insulin resistance, ovarian dysfunction, and androgen excess, further reinforcing the interconnected metabolic and endocrine disturbances characteristic of the syndrome. Together, these observations argue that PCOS is best conceptualized as a network disorder rather than a single-pathway disease; an interpretation that has direct implications for therapeutic development.

Current PCOS management remains largely symptom-oriented and frequently requires long-term, individualized regimens that may not comprehensively address both metabolic and reproductive abnormalities. Lifestyle intervention is foundational yet variably effective and difficult to sustain at scale. Pharmacotherapies, including insulin sensitizers, ovulation induction agents, and hormonal treatments, can be beneficial for specific goals but are often limited by incomplete efficacy across the PCOS spectrum, tolerability concerns, contraindications in certain patient groups, and the need for combination strategies to address multi-system disease manifestations [8]. In clinical practice, insulin sensitizers such as metformin are widely used to improve metabolic parameters, while ovulation induction agents (e.g., letrozole and clomiphene citrate) and combined oral contraceptives are commonly prescribed to regulate menstrual cycles and manage hyperandrogenism [9]. Lifestyle interventions, including dietary modification, weight reduction, and structured physical activity, remain first-line strategies and have been shown to improve insulin resistance, ovulatory function, and cardiometabolic risk profiles [10,11]. These limitations underscore a persistent translational gap: the need for interventions capable of engaging multiple PCOS-relevant nodes (metabolic and steroidogenic) with acceptable safety profiles and mechanistic clarity.

Multi-component phytotherapy is conceptually aligned with the network nature of PCOS. Spices and plant-derived preparations contain chemically diverse constituents that can, in principle, exert distributed effects on signaling, oxidative stress, inflammation, and hormone-related pathways. Cinnamon (*Cinnamomum* spp.) has been widely studied for glycemic and insulin-related effects, with mechanistic reports supporting roles in glucose metabolism and insulin signaling [12]. Nutmeg (*Myristica fragrans*) contains bioactive phenylpropanoids and lignans, and experimental studies have reported anti-inflammatory and metabolic modulatory potential, including effects on lipid synthesis and inflammatory processes in preclinical models [13]. Despite this promise, phytotherapeutic strategies for PCOS are often criticized for insufficient mechanistic anchoring to disease-relevant targets, limited integration with human omics data, and inconsistent experimental validation across both metabolic and ovarian contexts.

To address these gaps, we adopted an integrative workflow that deliberately moves from disease context to mechanism to functional validation. We first analyzed the ovarian-related transcriptomic dataset GSE137684, which includes normoandrogenic and hyperandrogenic PCOS subtypes, to capture molecular heterogeneity directly relevant to androgen status. Notably, the PCOS-versus-control differential analysis did not yield genes meeting the conventional multiple-testing thresholds (adjusted *p* < 0.05); we observed coordinated expression patterns among top-ranked candidates, consistent with subtle, distributed transcriptional shifts expected in heterogeneous syndromes. Building on these exploratory signals, functional enrichment and protein–protein interaction analyses prioritized steroidogenic and metabolic signaling architectures and identified a compact hub set—CYP19A1, CYP17A1, AKT1, ESR1, and MAPK1—that plausibly links androgen biosynthesis, estrogen signaling, and insulin-responsive pathways.

Collectively, our work is novel in explicitly linking exploratory human granulosa-cell transcriptomics to network-level target prioritization and then to dual-context in vitro validation (metabolic and ovarian), thereby strengthening mechanistic plausibility for spice-derived multi-target strategies in PCOS. Our central objective was to test the hypothesis that *C. burmannii* and *M. fragrans* can modulate interconnected metabolic and steroidogenic pathways relevant to PCOS. Specifically, we aimed to extract PCOS-relevant molecular signals from GSE137684 and prioritized functionally enriched targets, despite the absence of false discovery rate (FDR)-significant differentially expressed genes (DEGs), to define and rank multi-target interactions using network pharmacology and docking with emphasis on CYP19A1/CYP17A1/AKT1/ESR1/MAPK1, and to experimentally validate cytocompatibility and PCOS-relevant functional outputs in insulin-resistant adipocytes and DHEA-induced granulosa-like cells.

## 2. Materials and Methods

### 2.1. Preparation and Standardization of Plant Extracts

Dried bark of *Cinnamomum burmannii* and seeds of *Myristica fragrans* were authenticated and finely pulverized into homogeneous powders. Authentication of the plant materials was performed by botanists at the Integrated Laboratory of the Islamic State University of Sunan Kalijaga, Yogyakarta. Voucher specimens of the authenticated plant materials (*Cinnamomum burmannii* bark and *Myristica fragrans* seeds) were subsequently deposited in the Herbarium of the Faculty of Science and Technology at the same institution under reference number 051/LSB/REP/VI/2025. Each plant material (100 g) was extracted using 70% ethanol at a ratio of 1:10 (*w*/*v*) through maceration for 48 h at room temperature (25 ± 2 °C) with continuous agitation at 150 rpm to ensure optimal solvent penetration and compound diffusion. The resulting extracts were filtered through Whatman No. 1 filter paper and concentrated under reduced pressure at 40 °C using a rotary evaporator. The semi-solid extracts were subsequently freeze-dried to obtain stable crude extract powders.

Stock solutions were prepared at a concentration of 100 mg/mL in dimethyl sulfoxide (DMSO), vortexed thoroughly, and sterilized using a 0.22 μm syringe filter (Thermo Fisher Scientific, Waltham, MA, USA). Working solutions were freshly prepared by diluting stock solutions in culture medium to final concentrations of 6.25, 12.5, 25, 50, and 100 μg/mL. The final DMSO concentration was maintained at or below 0.1% (*v*/*v*) across all experimental conditions to avoid solvent-induced cytotoxicity. For combination treatments, cinnamon and nutmeg extracts were mixed at a fixed 1:1 ratio (*w*/*w*) prior to dilution, ensuring equivalent total concentrations relative to single-extract treatments.

### 2.2. In Silico Bioinformatics Analysis

#### 2.2.1. Transcriptomic Analysis of GSE137684

Gene expression profiling was performed using the publicly available dataset GSE137684 obtained from the NCBI GEO, which comprises ovarian-related samples from individuals diagnosed with polycystic ovary syndrome (PCOS) and healthy controls [14]. The dataset includes both normoandrogenic and hyperandrogenic PCOS phenotypes, enabling the exploration of transcriptional heterogeneity within PCOS. Raw expression data were downloaded as a series matrix file and processed using standard bioinformatics pipelines. Gene expression values were normalized to minimize technical variability across samples. Quality control was conducted through distributional assessment and principal component analysis (PCA). The PCA revealed that the first two principal components (PC1 and PC2) explained 44.8% and 12.5% of the total variance, respectively. Control samples exhibited relatively tight clustering, indicating consistency in baseline transcriptional profiles. In contrast, PCOS samples displayed broader dispersion, reflecting the intrinsic biological heterogeneity of the syndrome, particularly between normoandrogenic and hyperandrogenic subtypes. A small number of samples demonstrated distinct separation patterns, suggesting potential inter-individual variability.

#### 2.2.2. Metabolomic-Based Selection of Representative Phytochemicals

Representative phytochemicals from *C. burmannii* and *M. fragrans* were selected from triplicate metabolomic profiling datasets based on analytical reproducibility and signal intensity [15,16]. For each detected compound, mean peak area, standard deviation, and coefficient of variation (CV) were calculated across the three analytical replicates. Only compounds with CV values below 30% were retained to ensure acceptable repeatability. From cinnamon, the selected compounds included trans-cinnamaldehyde, cinnamaldehyde, coumarin, 4-methoxycinnamaldehyde, and 8-*p*-coumaroyl-3,4-dihydro-5,7-dihydroxy-4-phenylcoumarin. From nutmeg, the selected compounds included elemicin, safrole, terreusterpene C, licarin A, and 2′,4′,6′-trihydroxy-3′-prenyldihydrochalcone. These compounds were chosen as representative ligands for subsequent pharmacoinformatics analyses because they combined high analytical reproducibility with structurally diverse chemical classes, including phenylpropanoids, terpenoids, and related phytochemical derivatives.

Metabolomic profiling was conducted using a Thermo Scientific™ Vanquish™ Horizon Ultra High Performance Liquid Chromatography (UHPLC) system (Thermo Fisher Scientific, Waltham, MA, USA) coupled with a Thermo Scientific™ Accucore™ Phenyl Hexyl analytical column (100 mm × 2.1 mm internal diameter, 2.6 µm particle size) [17]. The chromatographic separation employed a binary mobile phase consisting of LC-MS grade water with 0.1% formic acid (solvent A) and LC-MS grade acetonitrile with 0.1% formic acid (solvent B). The flow rate was maintained at 0.3 mL/min over a total runtime of 25 min. The gradient elution program initiated at 5% solvent B, increased linearly to 90% within 16 min, held for 4 min, and subsequently re-equilibrated to the initial conditions until the end of the run.

High-resolution mass spectrometry (HRMS) analysis was performed using a Thermo Scientific™ Orbitrap™ Exploris 240 system (Thermo Fisher Scientific, Waltham, MA, USA) operating in Full MS/dd-MS^2^ acquisition mode under both positive and negative ionization conditions. The full MS resolution was set to 60,000 (FWHM), with a scan range spanning *m*/*z* 70–1000. Ionization was achieved using an Optamax™ NG Heated Electrospray Ionization (H-ESI) source (Thermo Fisher Scientific, Waltham, MA, USA), applying spray voltages of 3.5 kV in positive mode and 2.5 kV in negative mode, with auxiliary gas parameters optimized for stable ion generation.

Sample preparation involved the extraction of metabolites by adding 1 mL of HPLC-grade methanol to 50 mg of raw sample. The mixture was vortexed for 1 min, followed by sonication at 30 °C for 30 min, and subsequently centrifuged at 1400× *g* for 5 min. The resulting supernatant was filtered through a 0.2 µm nylon membrane prior to analysis.

Quality control (QC) procedures were implemented by generating pooled QC samples from equal aliquots (50 µL) of each extract, representing a comprehensive metabolite profile. These QC samples were periodically injected every 6–8 runs throughout the analytical sequence to monitor system stability, retention time reproducibility, and signal consistency. Instrument performance was further validated using a standard reference metabolite mixture prior to sample analysis. Data quality was confirmed through tight clustering of QC samples in principal component analysis (PCA) and by maintaining a CV below 30% across the QC-derived features.

Data processing and metabolite annotation were carried out using Thermo Scientific Compound Discoverer 3.3 software, supported by integrated databases including mzCloud, ChemSpider, and LipidMaps. Each sample was analyzed in triplicate (*n* = 3) under both ionization modes to ensure robust data acquisition for downstream chemometric analysis. Given the untargeted nature of this study, metabolite identification was performed at a putative level based on high mass accuracy (<5 ppm), isotopic pattern matching, and MS/MS spectral comparison against reference databases. Although MSI confidence levels were not explicitly assigned, the integration of high-resolution mass spectrometry with database-assisted annotation provided a reliable framework for comparative metabolomic and bioactivity analyses.

#### 2.2.3. Compound Target Prediction and PCOS-Related Gene Prioritization

The canonical SMILES structures of the selected phytochemicals were retrieved from the PubChem database and used as input for computational target prediction [18]. Putative human protein targets of each compound were predicted using SwissTargetPrediction, and only high-confidence targets were retained for subsequent analyses. In parallel, PCOS-relevant genes were compiled through the integration of transcriptomic findings from GSE137684 and literature-informed mechanistic relevance to steroidogenesis, hormone signaling, and metabolic dysregulation. Based on the convergence between transcriptomic trends, pathway enrichment, and disease biology, the final prioritized target set included SRD5A2, HDAC3, CYP19A1, PTGS2, ESR1, GSK3B, HMOX1, MAPK1, HSD17B3, CYP17A1, ESR2, and AKT1. The overlap between the predicted phytochemical targets and PCOS-relevant genes was used to identify shared candidate targets that could mechanistically link cinnamon and nutmeg bioactives to the molecular features of PCOS.

#### 2.2.4. Functional Enrichment and Protein–Protein Interaction Analysis

The overlapping targets were subjected to Gene Ontology (GO) and Kyoto Encyclopedia of Genes and Genomes (KEGG) enrichment analyses to characterize the biological processes, cellular components, molecular functions, and signaling pathways most relevant to the phytochemical–PCOS interaction network [19]. Enrichment analysis was performed using ShinyGO (version 0.85), and terms with adjusted FDR values below 0.05 were considered significantly enriched. GO analysis was categorized into Biological Process, Cellular Component, and Molecular Function, whereas KEGG analysis was used to identify pathways associated with steroid hormone biosynthesis, endocrine resistance, estrogen signaling, and related metabolic pathways. To further assess inter-target connectivity, protein–protein interaction (PPI) analysis was performed using the STRING database (version 12.0) restricted to *Homo sapiens* as well as a high-confidence interaction score threshold of 0.7. The resulting interaction network was imported into Cytoscape (version 3.10.1), where network topology parameters such as degree centrality, betweenness centrality, and closeness centrality were evaluated to identify hub genes. Through this procedure, CYP17A1, CYP19A1, AKT1, ESR1, and MAPK1 emerged as key interconnected nodes and were selected for molecular docking and experimental validation. This is consistent with the enrichment analysis results and hub-gene interpretation.

#### 2.2.5. Molecular Docking Analysis

Molecular docking simulations were carried out to evaluate the interaction potential of the selected phytochemicals with the major PCOS-related hub proteins identified by network analysis. Docking was performed using CB-Dock2, a cavity-detection-guided blind docking platform that integrates automatic pocket prediction and AutoDock Vina-based affinity scoring [20]. The three-dimensional structures of ligands were obtained from PubChem, while protein crystal structures were retrieved from the RCSB Protein Data Bank. The proteins used for docking were CYP17A1 (PDB ID: 3RUK), CYP19A1 (PDB ID: 3EQM), AKT1 (PDB ID: 4EJN), ESR1 (PDB ID: 3ERT), and MAPK1 (PDB ID: 6SLG). Crystallographic water molecules were removed automatically by the docking server before simulation. Docking scores were expressed as binding affinity values (kcal/mol), with more negative values indicating stronger predicted binding. Reference compounds were also included to provide comparative benchmarks, namely pioglitazone, flutamide, and aspirin, as reported in the docking results table. The top-ranked docking interactions were then interpreted in the context of PCOS-relevant steroidogenic and metabolic pathways.

### 2.3. In Vitro Experimental Study

#### 2.3.1. Cell Culture and Differentiation

Human granulosa-like tumor (KGN) cells were cultured in Dulbecco’s modified Eagle medium/Nutrient Mixture F-12 (DMEM/F-12; Gibco, Thermo Fisher Scientific, Waltham, MA, USA; 11995065) supplemented with 10% fetal bovine serum (FBS) and 1% penicillin–streptomycin [21]. Cells were maintained at 37 °C in a humidified incubator containing 5% CO_2_ and passaged at approximately 80% confluence using trypsin-EDTA. Only cells within passages 5–15 were used to ensure experimental consistency. Murine 3T3-L1 preadipocytes were cultured in high-glucose DMEM supplemented with 10% calf serum and 1% penicillin–streptomycin. Upon reaching full confluence (designated as day 0), adipogenic differentiation was initiated using a standard differentiation cocktail consisting of 0.5 mM 3-isobutyl-1-methylxanthine (IBMX), 1 μM dexamethasone, and 10 μg/mL insulin in DMEM supplemented with 10% FBS. After 48 h, the medium was replaced with DMEM containing 10% FBS and 10 μg/mL insulin. Subsequently, cells were maintained in DMEM with 10% FBS, with medium changes every two days until full differentiation was achieved at day 8–10, as confirmed by the morphological changes and lipid droplet accumulation.

#### 2.3.2. Experimental Design and Treatment Protocol

Both KGN cells and fully differentiated 3T3-L1 adipocytes were subjected to a standardized treatment scheme. Cells were divided into six experimental groups comprising the vehicle control (0.1% DMSO), induced model, induced cells treated with cinnamon extract, induced cells treated with nutmeg extract, induced cells treated with a cinnamon–nutmeg combination (1:1, *w*/*w*), and induced cells treated with a positive control compound. Extract treatments were administered at five concentrations (6.25, 12.5, 25, 50, and 100 μg/mL), while the positive control, pioglitazone, was applied at concentrations of 1, 2.5, 5, 10, and 20 μM. Following model induction, KGN cells were exposed to treatments for 24 h, whereas 3T3-L1 adipocytes were treated for 24 h after the induction of insulin resistance. All experiments were conducted in triplicate to ensure reproducibility and statistical validity.

#### 2.3.3. Induction of PCOS-like and Metabolic Dysfunction Models

To establish a PCOS-like cellular model, KGN cells were seeded at a density of 1 × 10^5^ cells per well in 6-well plates or 1 × 10^4^ cells per well in 96-well plates and allowed to adhere overnight. Cells were then exposed to dehydroepiandrosterone (DHEA) at a concentration of 100 μM for 24 h to induce hyperandrogenic and steroidogenic dysregulation characteristic of PCOS. Following induction, cells were gently washed with phosphate-buffered saline (PBS) to remove residual DHEA before subsequent treatment with plant extracts or control compounds. For the metabolic dysfunction model, fully differentiated 3T3-L1 adipocytes were incubated in high-glucose conditions (25 mM) supplemented with insulin at 100 nM for 24 h to induce insulin resistance. After induction, cells were treated with the respective plant extracts or positive control under identical experimental conditions.

#### 2.3.4. Cell Viability Assay

Cell viability was evaluated using the Cell Counting Kit-8 (CCK-8) assay. KGN and 3T3-L1 cells were seeded in 96-well plates at densities of 1 × 10^4^ and 8 × 10^3^ cells per well, respectively. Following treatment, 10 μL of CCK-8 reagent was added to each well containing 100 μL of culture medium and incubated for 2 h at 37 °C. Absorbance was measured at 450 nm using a microplate reader. Cell viability was calculated as a percentage relative to the vehicle control group, and only concentrations maintaining at least 80% viability were used for further analyses.

#### 2.3.5. Quantitative Real-Time PCR Analysis

Total RNA was extracted from treated cells using TRIzol reagent according to the manufacturer’s instructions [22]. RNA purity and concentration were determined spectrophotometrically by measuring absorbance at 260/280 nm. Complementary DNA (cDNA) was synthesized from 1 μg of total RNA using a reverse transcription kit. Quantitative real-time PCR (qRT-PCR) was performed using SYBR Green Master Mix (Applied Biosystems, Thermo Fisher Scientific, Waltham, MA, USA; A25742) in a real-time PCR system (QuantStudio™ 5, Applied Biosystems). Amplification conditions consisted of an initial denaturation step at 95 °C for 5 min, followed by 40 cycles of denaturation at 95 °C for 15 s and annealing/extension at 60 °C for 30 s. Gene expression levels were normalized to the housekeeping gene GAPDH and calculated using the 2^−ΔΔCt^ method. In KGN cells, the expression levels of CYP17A1, CYP19A1, ESR1, AKT1, and MAPK1 were analyzed to assess steroidogenic and signaling alterations. In 3T3-L1 adipocytes, AKT1, GSK3B, and GLUT4 were evaluated to determine metabolic and insulin signaling responses.

#### 2.3.6. Hormone Measurement

Cell culture supernatants from KGN cells were collected and centrifuged at 3000 rpm for 10 min to remove cellular debris. The concentrations of testosterone and estradiol were quantified using commercially available ELISA kits (Cayman Chemical, Ann Arbor, MI, USA; 501890 and 582701, respectively) according to the manufacturer’s protocols. Absorbance was measured at 450 nm, and hormone concentrations were calculated based on standard calibration curves.

#### 2.3.7. Glucose Uptake Assay

Glucose uptake in 3T3-L1 adipocytes was assessed using the fluorescent glucose analog 2-NBDG. After treatment, cells were serum-starved for 4 h to enhance insulin responsiveness and subsequently incubated with 2-NBDG at a concentration of 100 μM for 30 min at 37 °C. Cells were washed with PBS to remove excess dye, and fluorescence intensity was measured using a microplate reader at excitation and emission wavelengths of 485 nm and 535 nm, respectively. Results were normalized to cell number or protein content.

#### 2.3.8. Oil Red O Staining

Intracellular lipid accumulation in 3T3-L1 adipocytes was evaluated using Oil Red O staining [23]. Cells were washed with PBS and fixed with 4% paraformaldehyde for 30 min at room temperature. After fixation, cells were stained with freshly prepared Oil Red O working solution (Sigma-Aldrich, St. Louis, MO, USA; O1391) for 30 min and washed with distilled water to remove excess stain. Lipid droplets were visualized under a light microscope. For quantitative analysis, the retained dye was eluted using 100% isopropanol, and absorbance was measured at 520 nm. The results were expressed relative to the control group.

### 2.4. Statistical Analysis

All experiments were conducted in triplicate, and data were presented as the mean ± standard deviation (SD). Statistical analysis was performed using one-way analysis of variance (ANOVA), followed by Tukey’s post hoc test for multiple comparisons. A value of *p* < 0.05 was considered statistically significant.

## 3. Results

### 3.1. Transcriptomic Profiling and Identification of Candidate Genes in PCOS

**Figure 1:**
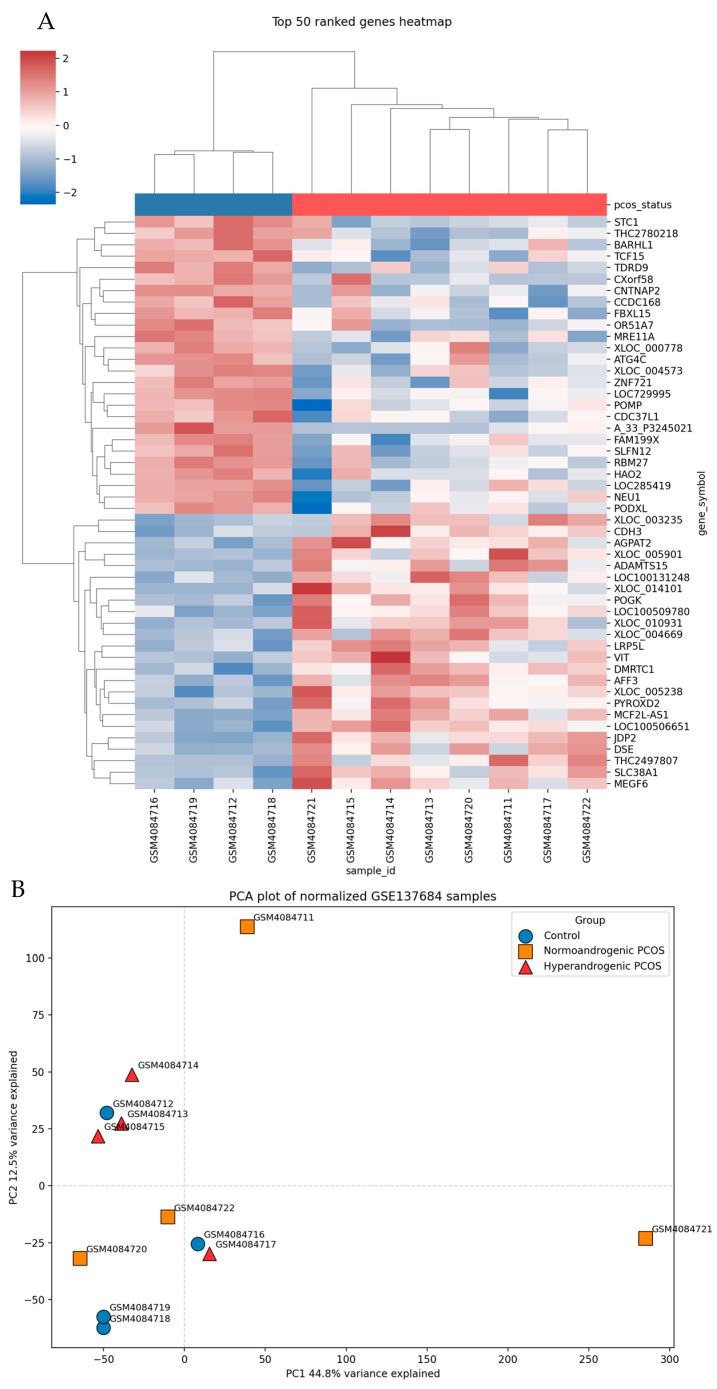
(**A**) Heatmap of the top 50 ranked genes showing differential expression patterns between PCOS and the control samples from the GSE137684 dataset. Hierarchical clustering revealed distinct gene expression profiles, with red indicating upregulation and blue indicating downregulation. (**B**) Principal component analysis (PCA) plot of normalized gene expression data from the GSE137684 dataset. Samples are grouped into control, normoandrogenic PCOS, and hyperandrogenic PCOS. PC1 (44.8%) and PC2 (12.5%) explain the major sources of variance, demonstrating partial separation between the PCOS subtypes and control samples.

Transcriptomic analysis of the GSE137684 dataset revealed distinct yet modest gene expression alterations between PCOS and the control samples. As shown in Figure 1, principal component analysis (PCA) demonstrated partial separation between groups, with PC1 and PC2 explaining 44.8% and 12.5% of the total variance, respectively. Control samples exhibited relatively tight clustering, whereas PCOS samples showed broader dispersion, reflecting the intrinsic heterogeneity of the syndrome, particularly between normoandrogenic and hyperandrogenic phenotypes. Consistent with this observation, hierarchical clustering of the top 50 ranked genes revealed discernible expression patterns distinguishing PCOS from the control samples. Although separation was not absolute, coordinated transcriptional shifts were evident, suggesting underlying biological perturbations associated with PCOS.

Despite these observable trends, no genes met the threshold for statistical significance after multiple-testing correction. Therefore, candidate genes were selected based on nominal significance (*p*-value < 0.01) and fold change criteria, as summarized in Table 1. Among the prioritized genes, several showed notable expression changes, including JDP2, STC1, AFF3, CDH3, IP6K3, POSTN, MFAP2, CASR, and NLRP2, which are associated with transcriptional regulation, metabolic processes, extracellular matrix remodeling, and inflammatory signaling. These findings suggest that PCOS is characterized by coordinated but subtle transcriptional reprogramming rather than strong single-gene effects.

### 3.2. Identification of Bioactive Compounds from Cinnamon and Nutmeg

**Figure 2:**
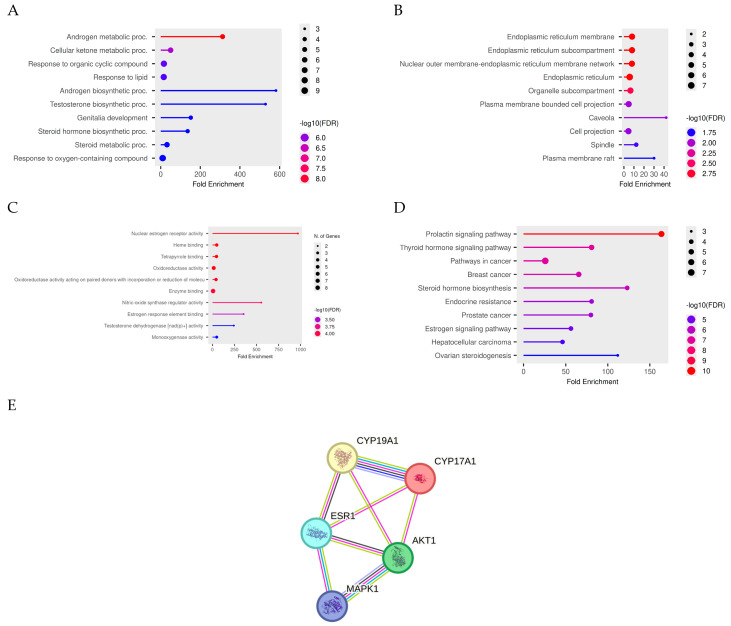
Integrated functional enrichment and protein interaction network of PCOS-related target genes. (**A**–**C**) Gene Ontology enrichment analyses highlighting biological processes, cellular components, and molecular functions; (**D**) KEGG pathway analysis revealing enrichment in steroid hormone biosynthesis, endocrine resistance, and estrogen signaling pathways; (**E**) protein–protein interaction network illustrating the interconnected roles of CYP19A1, CYP17A1, AKT1, ESR1, and MAPK1. Node size and color intensity correspond to enrichment significance and gene involvement.

Representative phytochemicals from *Cinnamomum burmannii* and *Myristica fragrans* were selected based on metabolomic reproducibility and signal intensity across triplicate analyses (Table 2). The selected compounds exhibited low coefficients of variation (CV < 30%), indicating high analytical consistency. Cinnamon-derived compounds were predominantly classified as phenylpropanoids, including trans-cinnamaldehyde, cinnamaldehyde, coumarin, and 4-methoxycinnamaldehyde, which are known for their bioactive properties in metabolic regulation. Nutmeg-derived compounds included elemicin, safrole, terreusterpene C, licarin A, and prenylated chalcone derivatives, representing a combination of phenylpropanoids, terpenoids, and other phytochemical classes. The diversity of chemical structures identified suggests the potential for multi-target interactions, supporting the rationale for a systems pharmacology approach to investigate their therapeutic relevance in PCOS. Integrated functional enrichment and protein interaction network of PCOS-related target genes were presented in Figure 2.

### 3.3. Molecular Docking Analysis of Bioactive Compounds

Molecular docking analysis revealed that several phytochemicals exhibited strong binding affinities toward key PCOS-related target proteins (Table 3). Among the tested compounds, 8-p-coumaroyl-3,4-dihydro-5,7-dihydroxy-4-phenylcoumarin demonstrated the highest binding affinity across multiple targets, with binding energies reaching −11.4 kcal/mol for CYP17A1 and −10.9 kcal/mol for AKT1.

Other compounds, such as licarin A and terreusterpene C, also exhibited favorable binding profiles comparable to reference drugs. While major compounds like cinnamaldehyde and coumarin showed moderate binding affinities, their consistent interaction across multiple targets suggests a multi-target mechanism rather than single-target potency.

Overall, the docking results indicate that cinnamon and nutmeg-derived compounds can interact with key proteins involved in steroidogenesis and metabolic signaling, supporting their potential therapeutic relevance in PCOS.

### 3.4. Effects on Cell Viability in Metabolic and Reproductive Models

**Figure 3:**
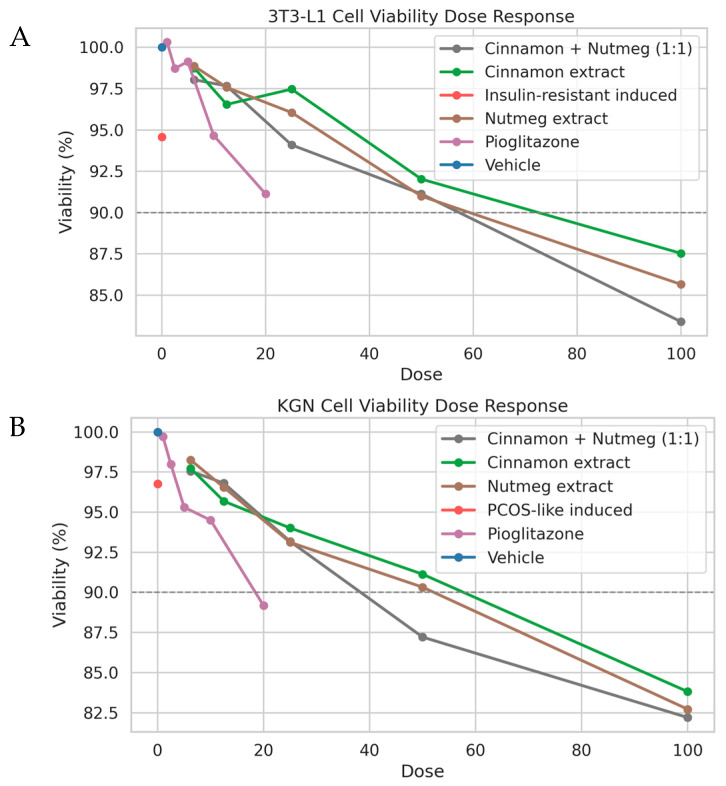
Concentration-dependent effects of cinnamon and nutmeg extracts on cellular viability in metabolic and reproductive PCOS models. (**A**) Insulin-resistant 3T3-L1 adipocytes and (**B**) DHEA-induced KGN cells were treated with individual extracts and their combination (1:1, *w*/*w*). Pioglitazone served as a reference drug. Cell viability remained above 80% across most tested concentrations, indicating acceptable cytocompatibility and suitability for subsequent functional assays.

The cytocompatibility of cinnamon and nutmeg extracts was evaluated in both insulin-resistant 3T3-L1 adipocytes and DHEA-induced KGN cells (Figure 3). Across all tested concentrations, cell viability remained above 80%, indicating that the extracts did not exert significant cytotoxic effects.

A gradual decrease in viability was observed at higher concentrations, particularly for combination treatments; however, the values remained within the acceptable limits for further functional assays. These findings confirm that the selected concentrations are suitable for downstream mechanistic investigations.

### 3.5. Modulation of Metabolic Function and Insulin Signaling in Adipocytes

**Figure 4:**
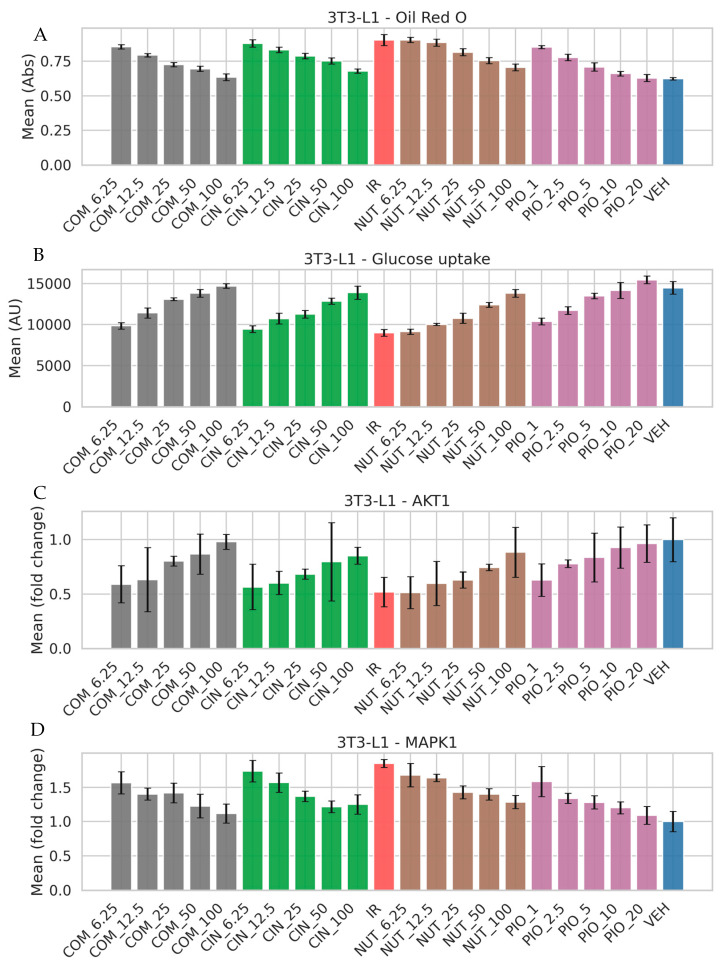
Modulatory effects of cinnamon and nutmeg extracts on metabolic dysfunction and signaling pathways in insulin-resistant 3T3-L1 adipocytes. Treatment with individual extracts and their combination (1:1, *w*/*w*) reduced lipid accumulation (**A**) and enhanced glucose uptake (**B**) while restoring key insulin signaling markers AKT1 (**C**) and MAPK1 (**D**). Pioglitazone (PIO) was used as a reference drug. These findings suggest improved metabolic function and partial recovery of insulin signaling under extract treatment. COM_6.25–100, CIN_6.25–100, and NUT_6.25–100 denote combination (1:1, *w*/*w*), cinnamon, and nutmeg extracts at 6.25–100 µg/mL, respectively; PIO_1–20 indicates pioglitazone (1–20 µM); DHEA represents induced cells (100 µM), and VEH denotes vehicle control (0.1% DMSO). Significant differences between vehicle (VEH) and insulin-resistant (IR) groups were observed for glucose uptake (*p* = 9.37 × 10^−5^), Oil Red O staining (*p* = 5.60 × 10^−4^), AKT1 expression (*p* = 0.00997), and MAPK1 expression (*p* = 5.12 × 10^−4^).

In insulin-resistant 3T3-L1 adipocytes, treatment with cinnamon and nutmeg extracts significantly improved the metabolic parameters (Figure 4). Oil Red O staining demonstrated a reduction in intracellular lipid accumulation, indicating attenuation of lipid storage. In parallel, glucose uptake was markedly enhanced following treatment, suggesting improved insulin sensitivity. At the molecular level, the expression of AKT1 and MAPK1 was partially restored, indicating the reactivation of key insulin signaling pathways. Notably, the combination treatment exhibited more consistent improvements across multiple endpoints compared to individual extracts, suggesting a potential synergistic effect.

### 3.6. Regulation of Hormonal Balance and Steroidogenic Pathways in KGN Cells

**Figure 5:**
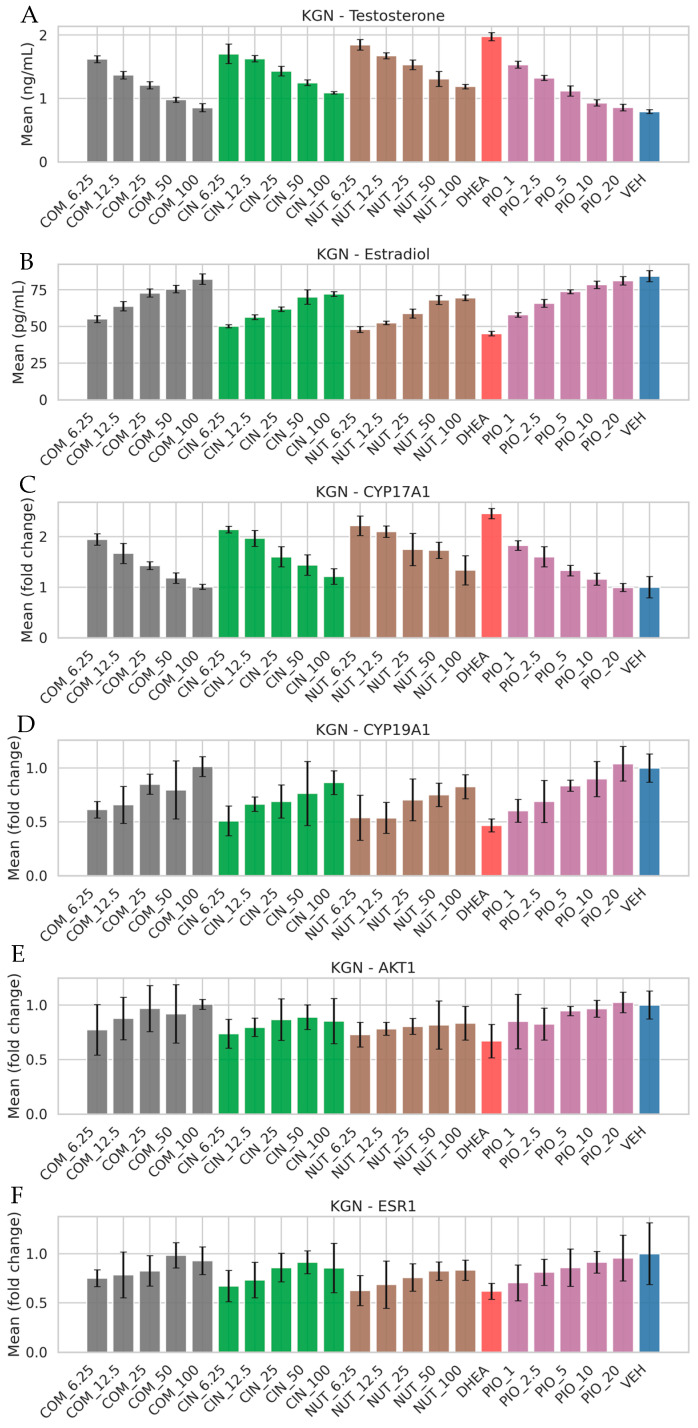
Modulatory effects of cinnamon and nutmeg extracts on endocrine dysfunction and steroidogenic pathways in PCOS-like KGN cells. Treatment reduced testosterone levels (**A**) and restored estradiol production (**B**), accompanied by the downregulation of CYP17A1 and upregulation of CYP19A1, indicating normalization of steroidogenesis. Additionally, key signaling molecules AKT1 and ESR1 were partially restored (**C**–**F**), suggesting improved cellular responsiveness to hormonal regulation. Pioglitazone (PIO) served as a reference compound. COM_6.25–100, CIN_6.25–100, and NUT_6.25–100 denote combination (1:1, *w*/*w*), cinnamon, and nutmeg extracts at 6.25–100 µg/mL, respectively; PIO_1–20 indicates pioglitazone (1–20 µM); DHEA represents induced cells (100 µM), and VEH denotes vehicle control (0.1% DMSO). Compared with vehicle (VEH), DHEA significantly altered estradiol (*p* = 4.76 × 10^−5^) and testosterone levels (*p* = 1.95 × 10^−6^). Gene expression analysis showed significant changes in AKT1 (*p* = 0.0172), CYP17A1 (*p* = 1.52 × 10^−4^), CYP19A1 (*p* = 0.00150), and MAPK1 (*p* = 6.65 × 10^−4^), while ESR1 was not significant (*p* = 0.0896).

In DHEA-induced KGN cells, treatment with cinnamon and nutmeg extracts significantly modulated endocrine function (Figure 5). Testosterone levels were reduced, while estradiol production was restored, indicating the correction of hyperandrogenism and hormonal imbalance.

At the molecular level, expression of CYP17A1 (androgen synthesis) was downregulated, whereas CYP19A1 (aromatase) was upregulated, suggesting normalization of steroidogenesis. Additionally, the AKT1 and ESR1 expression levels were partially restored, indicating improved signaling associated with metabolic and reproductive regulation. These results demonstrate that the extracts not only modulate metabolic dysfunction but also directly influence hormonal and steroidogenic pathways relevant to PCOS.

## 4. Discussion

**Figure 6:**
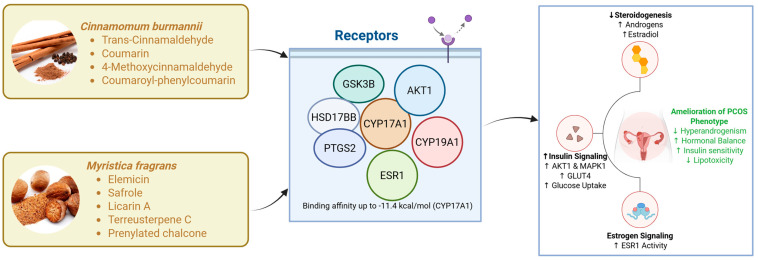
Schematic representation of the multi-target mechanism of action of Cinnamomum burmannii and Myristica fragrans in polycystic ovary syndrome (PCOS). Bioactive phytochemicals interact with key hub proteins (CYP17A1, CYP19A1, AKT1, ESR1, MAPK1), leading to the modulation of steroidogenesis, insulin signaling, and estrogen pathways. These effects result in reduced androgen production, increased estradiol synthesis, improved glucose uptake, and decreased lipid accumulation, ultimately contributing to the amelioration of PCOS-related metabolic and endocrine dysfunctions.

In this integrative study, we combined exploratory transcriptomics from GSE137684 with network pharmacology, molecular docking, and two complementary in vitro models to evaluate whether *Cinnamomum burmannii* and *Myristica fragrans* can modulate interconnected metabolic and steroidogenic features of PCOS (Figure 6). Although the GSE137684 comparison yielded no FDR-significant DEGs, PCA (PC1 44.8%, PC2 12.5%) indicated partial group structure, and downstream enrichment/PPI prioritized a coherent hub set (CYP19A1, CYP17A1, AKT1, ESR1, MAPK1). Guided by these hubs, docking suggested plausible multi-target binding (best reported ligand: −11.4 kcal/mol for CYP17A1; −10.9 kcal/mol for AKT1), and cellular assays showed cytocompatibility (≥80% viability) alongside improvements in lipid accumulation, glucose uptake, insulin-signaling markers, and steroidogenic/hormonal readouts.

### 4.1. Interpreting the Transcriptomic Signals in the Context of PCOS Heterogeneity

To identify DEGs, statistical comparisons between PCOS and control groups were performed using a linear modeling approach. Genes were initially ranked based on fold change and statistical significance. However, after adjustment for multiple testing, no genes met the conventional threshold for statistical significance (adjusted *p* < 0.05). This finding suggests that transcriptional alterations in this dataset are relatively subtle and may be influenced by biological variability, sample size, and the heterogeneous nature of PCOS. Despite the absence of statistically significant DEGs under stringent correction, exploratory analysis of the top-ranked genes revealed discernible expression patterns. A heatmap of the top 50 ranked genes demonstrated partial segregation between PCOS and the control samples, indicating the presence of coordinated transcriptional shifts. Several genes exhibited consistent upregulation or downregulation trends in PCOS, suggesting underlying biological processes that may contribute to disease pathophysiology. Notably, genes associated with metabolic regulation, extracellular matrix remodeling, immune response, and cellular signaling appeared among the top candidates.

A key observation from the GSE137684 analysis was the combination of (i) partial separation by PCA and (ii) absence of genes surviving multiple-testing correction. We interpreted this pattern as consistent with PCOS being a biologically heterogeneous syndrome with modest, distributed transcriptional perturbations rather than a single dominant gene-expression signature. The PCA structure (PC1 44.8%, PC2 12.5%) supports the presence of systematic variation across samples, while the lack of FDR-significant DEGs highlights limited statistical power and/or high inter-individual variability—both common issues in the transcriptomic analyses of heterogeneous endocrine–metabolic conditions. Importantly, we avoided overinterpretation by treating the differentially ranked genes as nominal candidates rather than definitive biomarkers.

Even within this exploratory framing, the top-ranked candidates (Table 1) provide biologically plausible “anchors” for mechanism-building. Several nominal candidates map to broad processes often implicated in PCOS pathophysiology, including transcriptional regulation (e.g., JDP2, AFF3), extracellular matrix organization and tissue remodeling (e.g., POSTN, MFAP2), inflammatory/innate immune signaling (e.g., NLRP2, DDX58), and metabolic regulation/stress responses (e.g., STC1, MT1G). While these associations are interpretative and require targeted validation, they strengthen the rationale for pathway- and network-level analyses rather than reliance on single-gene effects.

### 4.2. Network-Level Prioritization Converges on Steroidogenesis–Metabolism Signaling Hubs

To move from exploratory transcript-level signals to mechanistic hypotheses, we used enrichment analyses and PPI topology to identify convergent biology. The resulting hub set—CYP19A1, CYP17A1, AKT1, ESR1, and MAPK1—captured the conceptual “bridge” between ovarian steroidogenesis and systemic/metabolic signaling. CYP17A1 and CYP19A1 are central enzymes governing androgen synthesis and aromatization, respectively, and thus jointly shape the testosterone–estradiol balance [24]. AKT1 and MAPK1 are canonical signaling nodes that integrate metabolic cues with cellular growth and differentiation programs [25]; ESR1 provides a direct conduit for estrogen-responsive transcriptional regulation. This convergence is consistent with current views that PCOS emerges from tightly interlinked endocrine and metabolic dysregulation rather than isolated pathway defects [26]. Notably, the use of network prioritization mitigates (but does not eliminate) the limitations of weak single-gene significance by focusing on coherent, functionally enriched modules.

### 4.3. Docking Supports Multi-Target Plausibility but Requires Biochemical Confirmation

Within this target framework, docking was used as a hypothesis-generating step to assess whether cinnamon- and nutmeg-derived phytochemicals could plausibly engage the prioritized protein set. The strongest binding energies in our summary were observed for a top-ranked ligand (−11.4 kcal/mol for CYP17A1; −10.9 kcal/mol for AKT1), suggesting potential direct interactions with both a steroidogenic enzyme and a metabolic signaling kinase. These values are supportive of multi-target feasibility, particularly when interpreted alongside the chemical diversity of plant extracts. However, docking remains an in silico approximation that does not confirm target engagement, affinity in physiological settings, or functional inhibition/activation. As details of the broader docking matrix and interaction profiles are not fully reported, the docking results should therefore be interpreted as supportive rather than definitive evidence.

### 4.4. Functional Validation in Metabolic and Ovarian Cellular Models Aligns with the Hub Biology

The experimental design evaluated two distinct but disease-relevant cellular contexts: insulin-resistant 3T3-L1 adipocytes (metabolic axis) and DHEA-induced KGN cells (ovarian steroidogenic axis). First, cytocompatibility profiling (Figure 3) showed that viability remained ≥80% across the tested concentrations, supporting that downstream functional changes are less likely to be artifacts of overt toxicity. In insulin-resistant 3T3-L1 adipocytes (Figure 4), extract treatments reduced Oil Red O staining (interpreted as reduced intracellular lipid accumulation) and increased glucose uptake, consistent with improved metabolic handling under insulin-resistant conditions. At the signaling level, partial restoration of AKT1 and MAPK1 expression provides mechanistic alignment with the hub network and suggests that the extracts may act—directly or indirectly—on nodes that govern insulin responsiveness and cellular metabolic programs [27]. While we measured transcript-level changes rather than phosphorylation or enzymatic activity, the concordance between improved glucose uptake and modulation of AKT1/MAPK1 supports the plausibility of pathway rebalancing.

In DHEA-induced KGN cells (Figure 5), treatments decreased testosterone and increased estradiol, accompanied by the downregulation of CYP17A1 and upregulation of CYP19A1. This pattern coheres with a shift away from androgen excess toward increased aromatization capacity—a central therapeutic objective in hyperandrogenic PCOS phenotypes. Partial restoration of AKT1 and ESR1 further suggests that metabolic signaling and estrogen responsiveness may be simultaneously improved [28]. Importantly, these outcomes mirror the logic of the transcriptomics-guided hub selection: the same nodes identified by enrichment/PPI are the ones that shift in the expected direction under extract treatment.

Across endpoints, the cinnamon–nutmeg combination often appeared more consistent than individual extracts. While we did not perform formal synergy quantification (e.g., combination index analysis), the pattern is consistent with the expectation that multi-component mixtures can exert complementary actions across multiple targets and pathways. This is particularly relevant in PCOS, where combined metabolic and steroidogenic modulation may be necessary to achieve meaningful phenotype-level improvement.

### 4.5. Relationship to Existing Literature and Therapeutic Context

Prior literature (primary sources not specified in this draft) has frequently highlighted the limitations of single-pathway interventions in PCOS and the need to address both metabolic and reproductive features [29]. Evidence also suggests that cinnamon-derived constituents may influence glucose metabolism and insulin-related pathways, whereas nutmeg contains bioactives with reported metabolic and anti-inflammatory potential in non-PCOS settings [12,13]. Our study extends these lines of inquiry by anchoring target selection in the human ovarian transcriptomic context and by experimentally testing both metabolic and steroidogenic readouts in parallel. This design strengthens biological plausibility for multi-target phytotherapy while also clarifying that the transcriptomic component is exploratory rather than definitive.

### 4.6. Strengths

The major strengths of this work are the integrative, hypothesis-to-validation pipeline and the alignment of in silico prioritization with functional readouts. We linked exploratory transcriptomic structure to functionally enriched hubs (CYP19A1/CYP17A1/AKT1/ESR1/MAPK1), used docking to assess compatibilities with phytochemicals, and then tested predicted pathway directions in two disease-relevant cellular models. The parallel assessment of lipid handling, glucose uptake, hormone secretion, and steroidogenic/signaling gene expression provides internally consistent evidence for multi-axis modulation.

### 4.7. Limitations and Alternative Explanations

Several limitations constrain interpretation. First, the transcriptomic analysis yielded no FDR-significant DEGs, so downstream candidate selection is nominal and exploratory, increasing the risk of false positives and pathway overfitting. Second, the sample size and clinical covariates of the transcriptomic cohort are not specified here, limiting the assessment of confounding and generalizability. Third, docking results are incomplete in the present summary beyond the strongest reported ligand–target pairs, and docking does not confirm binding or functional modulation. Fourth, we tested crude extracts rather than isolated compounds, so the active constituents, their relative contributions, and potential antagonistic interactions remain unidentified. Fifth, our mechanistic assays rely largely on transcript-level measures; protein abundance, phosphorylation states (e.g., p-AKT, p-ERK), and functional enzyme activity (CYP17A1/CYP19A1) were not directly quantified. Finally, in vitro models (insulin-resistant 3T3-L1; DHEA-induced KGN) capture only selected aspects of PCOS and cannot substitute for in vivo endocrine physiology or clinical outcomes.

### 4.8. Implications and Future Directions

Despite these constraints, the study provides a practical framework for transcriptomics-guided network pharmacology in PCOS: where single-gene significance is weak, pathway coherence and hub convergence can still generate testable hypotheses. Next steps should prioritize (i) validation of the hub network in larger and/or independent transcriptomic cohorts (including meta-analytic approaches and phenotype-stratified analyses), (ii) target-focused assays that confirm functional engagement (CYP17A1 and aromatase activity assays; ESR1 reporter activity; AKT/MAPK phosphorylation profiling), and (iii) isolation and recombination of key constituents to quantify synergy and determine minimal active combinations. ADMET profiling and standardization strategies will be essential for translational credibility, followed by validation in in vivo PCOS models, and ultimately clinical evaluation to assess efficacy across PCOS phenotypes and safety with longer-term exposure.

## 5. Conclusions

In conclusion, this study provides integrative evidence that *Cinnamomum burmannii* and *Myristica fragrans* exert multi-target modulatory effects on key metabolic and steroidogenic pathways implicated in polycystic ovary syndrome (PCOS). By leveraging exploratory transcriptomic signals from the GSE137684 dataset, we identified a functionally coherent network centered on CYP17A1, CYP19A1, AKT1, ESR1, and MAPK1, highlighting the interconnected nature of androgen biosynthesis, estrogen signaling, and metabolic regulation in PCOS. Subsequent network pharmacology and molecular docking analyses demonstrated that bioactive phytochemicals from both plants can interact with these targets with favorable binding affinities, supporting a systems-level mechanism of action.

Importantly, experimental validation in both insulin-resistant adipocytes and DHEA-induced granulosa-like cells confirmed that these extracts are cytocompatible and capable of improving key disease-relevant endpoints. The treatments enhanced glucose uptake, reduced lipid accumulation, restored insulin signaling, and normalized steroidogenic imbalance by reducing testosterone levels and promoting estradiol production. The combination of cinnamon and nutmeg consistently showed more pronounced and stable effects across multiple parameters, suggesting potential synergistic interactions.

Collectively, these findings reinforce the concept of PCOS as a network disorder requiring multi-target therapeutic strategies. This study bridges human transcriptomic insights with mechanistic and functional validation, providing a biologically grounded framework for the development of plant-derived, multi-component interventions. Future studies should further investigate dose optimization, long-term efficacy, and clinical translation to validate the therapeutic potential of these phytochemicals in diverse PCOS phenotypes.

## Figures and Tables

**Table 1:** Nominally prioritized candidate genes in the PCOS versus control comparison from the GSE137684 dataset.

Gene Symbol	log2 Fold Change	Direction	*p*-Value	Adjusted *p*-Value
JDP2	1.323	Upregulated	3.79 × 10^−5^	0.6917
STC1	−1.341	Downregulated	4.46 × 10^−4^	0.7908
AFF3	1.287	Upregulated	5.45 × 10^−4^	0.7908
CNTNAP2	−1.886	Downregulated	7.03 × 10^−4^	0.7908
HAO2	−1.197	Downregulated	8.71 × 10^−4^	0.7908
DMRTC1	1.778	Upregulated	1.19 × 10^−3^	0.7908
ADAMTS15	1.288	Upregulated	1.44 × 10^−3^	0.7908
MEGF6	2.826	Upregulated	1.93 × 10^−3^	0.7908
PODXL	−1.611	Downregulated	2.23 × 10^−3^	0.7908
VIT	2.171	Upregulated	2.28 × 10^−3^	0.7908
CDH3	1.742	Upregulated	2.29 × 10^−3^	0.7908
C2CD4B	−1.714	Downregulated	2.43 × 10^−3^	0.7908
IP6K3	1.491	Upregulated	2.95 × 10^−3^	0.7908
POSTN	−2.691	Downregulated	3.64 × 10^−3^	0.7908
MFAP2	2.260	Upregulated	3.88 × 10^−3^	0.7908
CASR	2.598	Upregulated	3.92 × 10^−3^	0.7908
NLRP2	−2.046	Downregulated	4.26 × 10^−3^	0.7908
MT1G	−1.716	Downregulated	4.89 × 10^−3^	0.7908
DDX58	1.124	Upregulated	6.02 × 10^−3^	0.7908
SLC37A1	1.026	Upregulated	6.52 × 10^−3^	0.7908
MCF2	1.351	Upregulated	6.64 × 10^−3^	0.7908
MCF2L-AS1	2.720	Upregulated	2.05 × 10^−5^	0.6917
OR51A7	−1.078	Downregulated	2.09 × 10^−3^	0.7908
DKFZp761E198	1.638	Upregulated	2.44 × 10^−3^	0.7908
JSRP1	−1.694	Downregulated	2.96 × 10^−3^	0.7908
PPFIA2	−1.589	Downregulated	3.33 × 10^−3^	0.7908
RHOXF2	1.158	Upregulated	3.54 × 10^−3^	0.7908
WDR86	1.659	Upregulated	3.83 × 10^−3^	0.7908
ARMCX3	−1.104	Downregulated	4.05 × 10^−3^	0.7908
ADARB2	1.147	Upregulated	4.25 × 10^−3^	0.7908
DOK7	1.215	Upregulated	4.40 × 10^−3^	0.7908
CCDC160	1.146	Upregulated	5.10 × 10^−3^	0.7908
NTNG1	1.917	Upregulated	5.36 × 10^−3^	0.7908
MYH11	3.068	Upregulated	5.48 × 10^−3^	0.7908
ABLIM1	1.075	Upregulated	5.71 × 10^−3^	0.7908
TMC5	1.870	Upregulated	5.90 × 10^−3^	0.7908
SYNE2	1.015	Upregulated	5.99 × 10^−3^	0.7908
NRCAM	−1.644	Downregulated	6.09 × 10^−3^	0.7908
SPRR2F	2.117	Upregulated	6.23 × 10^−3^	0.7908
WNK2	1.051	Upregulated	6.45 × 10^−3^	0.7908
GAB2	1.238	Upregulated	6.65 × 10^−3^	0.7908
ENPP3	−1.200	Downregulated	6.72 × 10^−3^	0.7908
KLK3	1.047	Upregulated	6.77 × 10^−3^	0.7908
HTRA3	−1.294	Downregulated	6.91 × 10^−3^	0.7908
SPRR2C	1.423	Upregulated	7.24 × 10^−3^	0.7908
MPHOSPH6	−1.198	Downregulated	7.26 × 10^−3^	0.7908
RAB39B	1.027	Upregulated	7.38 × 10^−3^	0.7908
DENND2A	1.404	Upregulated	7.70 × 10^−3^	0.7908
IVNS1ABP	1.132	Upregulated	7.91 × 10^−3^	0.7908
SBK1	1.640	Upregulated	8.04 × 10^−3^	0.7908
DEFB105B	−1.149	Downregulated	8.45 × 10^−3^	0.7908
TP53I11	1.121	Upregulated	8.90 × 10^−3^	0.7908
ANKRD55	1.938	Upregulated	8.95 × 10^−3^	0.7908
UBXN10	−1.066	Downregulated	8.99 × 10^−3^	0.7908
TEX21P	−1.049	Downregulated	9.02 × 10^−3^	0.7908
PRKCQ	1.615	Upregulated	9.06 × 10^−3^	0.7908
FTCD	−1.620	Downregulated	9.11 × 10^−3^	0.7908
GPX3	1.824	Upregulated	9.30 × 10^−3^	0.7908
THC2670340	2.269	Upregulated	9.39 × 10^−3^	0.7908
ATP9A	1.165	Upregulated	9.39 × 10^−3^	0.7908
GAGE7	1.357	Upregulated	9.84 × 10^−3^	0.7908

Note. Genes were prioritized using the criteria *p*-value < 0.01 and absolute log2 fold change > 1. Anonymous transcript identifiers were excluded. No genes remained significant after multiple-testing correction; therefore, these genes should be interpreted as nominal candidate genes rather than FDR-significant differentially expressed genes.

**Table 2:** Selection of representative bioactive phytochemicals from cinnamon and nutmeg based on reproducibility and intensity across triplicate metabolomics datasets.

Samples	Compound Name	Formula	*m*/*z*	RT	Mean Area	Area SD	CV (%)	Biological Class
Cinnamon	trans-Cinnamaldehyde	C_9_H_8_O	133.065	6.722	14,622,613,950.88	267,640,887.84	1.83	Phenylpropanoid
	Cinnamaldehyde	C_9_H_8_O	133.065	6.654	14,611,647,335.2	264,612,140.51	1.81	Phenylpropanoid
	Coumarin	C_9_H_6_O_2_	147.0443	5.446	13,178,558,365.12	105,810,879.82	0.8	Phenylpropanoid
	4-Methoxycinnamaldehyde	C_10_H_10_O_2_	163.0757	7.584	184,266,080.11	981,915.64	0.53	Phenylpropanoid
	8-p-Coumaroyl-3,4-dihydro-5,7-dihydroxy-4-phenylcoumarin	C_24_H_18_O_6_	403.1185	6.03	22,786,661.84	310,601.76	1.36	Phenylpropanoid
Nutmeg	Elemicin	C_12_H_16_O_3_	209.1176	8.796	12,444,803,692.6	1,400,800,734.33	11.26	Phenylpropanoid
	Safrole	C_10_H_10_O_2_	163.0757	11.154	1,783,251,951.09	42,719,436.46	2.4	Phenylpropanoid
	Terreusterpene C	C_26_H_36_O_9_	491.2273	13.303	11,598,044.48	1,020,783.05	8.8	Terpenoid
	Licarin A	C_20_H_22_O_4_	327.1599	10.794	11,467,997,460.38	262,265,374.78	2.29	Other phytochemical
	2′,4′,6′-Trihydroxy-3′-prenyldihydrochalcone	C_20_H_22_O_4_	327.1599	10.86	11,407,968,648.37	257,655,843.34	2.26	Other phytochemical

Mean intensity, standard deviation (SD), and coefficient of variation (CV) were calculated across triplicates. Compounds with CV > 30% were excluded to ensure reproducibility.

**Table 3:** Molecular docking binding affinities (kcal/mol) of selected bioactive compounds from *Cinnamomum burmannii* and *Myristica fragrans* against key PCOS-related target proteins (CYP17A1, CYP19A1, AKT1, ESR1, and MAPK1).

	CYP17A1 (PDB ID:3RUK)	CYP19A1 (PDB ID: 3EQM)	AKT1 (PDB ID: 4EJN)	ESR1 (PDB ID: 3ERT)	MAPK1 (PDB ID: 6SLG)
Pioglitazone/Control	−8.5	−8.4	−9.3	−8.4	−8.0
Flutamide/Control	−7.1	−7.3	−7.8	−7.7	−6.9
Aspirin/Control	−5.9	−6.0	−6.6	−6.4	−5.7
trans-Cinnamaldehyde	−5.9	−6.0	−6.3	−5.9	−5.3
Cinnamaldehyde	−6.1	−5.8	−6.1	−5.8	−5.2
Coumarin	−6.6	−6.4	−7.4	−6.6	−5.6
4-Methoxycinnamaldehyde	−6.0	−5.7	−6.4	−5.6	−5.4
8-p-Coumaroyl-3,4-dihydro-5,7-dihydroxy-4-phenylcoumarin	−11.4	−9.9	−10.9	−8.5	−9.2
Elemicin	−5.8	−5.8	−6.4	−6.0	−5.2
Safrole	−6.2	−5.8	−6.6	−6.1	−5.5
Terreusterpene C	−9.0	−8.4	−8.6	−7.9	−8.3
Licarin A	−8.5	−8.9	−9.7	−7.6	−7.9
2′,4′,6′-Trihydroxy-3′-prenyldihydrochalcone	−9.5	−7.9	−9.1	−8.3	−6.9

## Data Availability

The original contributions presented in this study are included in the article. The datasets used and/or analyzed during the current study are available from the corresponding authors on reasonable request. Further inquiries can be directed to the corresponding authors.
